# Supplementary material for: Diet‐induced plasticity of life‐history traits and gene expression in outbred Drosophila melanogaster population
Source: Ecol Evol. 2024 Feb 9;14(2):e10976. doi: 10.1002/ece3.10976 (PMC10857930; doi:10.1002/ece3.10976)
Supplement: Supplementary file 1 — Data S1. [file ECE3-14-e10976-s002.docx]

**Supplementary Online Materials** for

Diet-induced plasticity of life-history traits and gene expression in outbred *Drosophila melanogaster* population

Text S1: Wing length measurement protocol

To understand the effect of diet on body size, we measured the wing size of the flies as a proxy. On the 12^th^ day post egg collection, flies were separated under CO_2_ anesthesia and arbitrarily one of the two wings were clipped off for 30 individuals per sex. The wings were then mounted on a microscopic slide using a drop of polyethylene glycol (PEG) and imaged. The length of the vein between distance between the anterior cross vein (ACV) and the third longitudinal vein (L3) was measured using ImageJ as a standard metric for wing size.

| **Table S1. Recipes to make isocaloric diets** (714KCal). The flies get carbohydrates from sugar (energy = 3.87KCal/gm) and corn (energy = 3.33KCal/gm) whereas they get proteins and carbohydrates from yeast (energy = 3.54kCal/gm). The P:C ratio has been calculated considering the total protein and the total carbohydrate that has been contributed by each diet component. |
| --- |
| 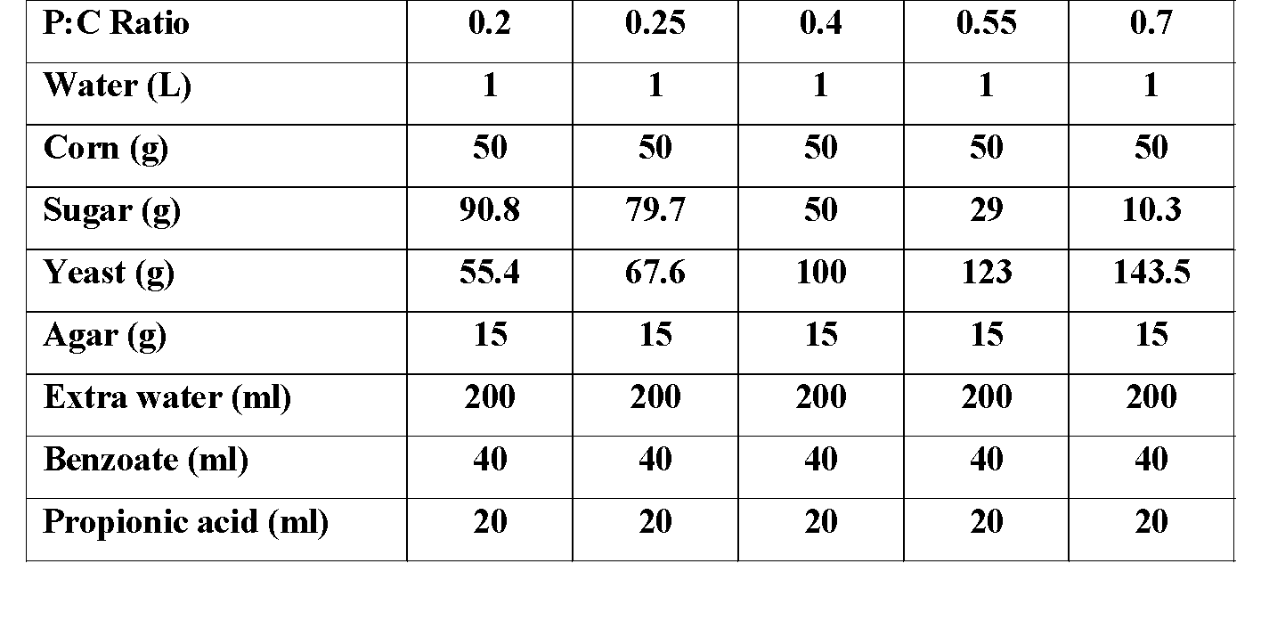 |
| *Per gram, yeast contains 0.37g of carbohydrates and 0.48g of protein, while corn contains 0.78g of carbohydrates and 0.07g of protein. |

The following tables are attached as separate Excel files for better data representations.

**Table S2. Complete List of Expressed Transcripts Across Samples**

**Table S3. Differential Transcript Expression Statistics: P:C = 0.25 Diet vs. Baseline (P:C = 0.4) Diet**

**Table S4. Differential Transcript Expression Statistics: P:C = 0.55 Diet vs. Baseline (P:C = 0.4) Diet**

**Table S5. Differential Transcript Expression Statistics: P:C = 0.7 Diet vs. Baseline (P:C = 0.4) Diet**

**Table S6. GO Analysis of Transcripts Upregulated in P:C = 0.25 Diet vs. Baseline (P:C = 0.4) Diet.**

**Table S7. GO Analysis of Transcripts Upregulated in P:C = 0.7 Diet vs. Baseline (P:C = 0.4) Diet.**

| **Table S8. Effect size of differential mean locomotor activity of old male flies reared on experimental diets** |
| --- |
| \| **Groups** \| **Cohen's *d*** \| **Inference** \| \| --- \| --- \| --- \| \| P:C 0.2 - P:C 0.25 \| 0.2 \| Small \| \| P:C 0.2 - P:C 0.4 \| 0.38 \| Small \| \| P:C 0.2 - P:C 0.55 \| 0.15 \| n.d. \| \| **P:C 0.2 - P:C 0.7** \| **0.57** \| **Medium** \| \| **P:C 0.25 - P:C 0.4** \| **0.58** \| **Medium** \| \| P:C 0.25 - P:C 0.55 \| 0.35 \| Small \| \| **P:C 0.25 - P:C 0.7** \| **0.82** \| **Large** \| \| P:C 0.4 - P:C 0.55 \| 0.24 \| Small \| \| P:C 0.4 - P:C 0.7 \| 0.15 \| n.d. \| \| P:C 0.55 - P:C 0.7 \| 0.41 \| Small \| |
|  |
| d < 0.2: n.d. or no difference, < d < 0.5: small, 0.5 < d < 0.8: medium), d > 0.8: large |

| **Table S9. Analysis of Deviance Table (Type III tests) comparing survival curves in lifespan assay using Cox-Proportional hazard model followed by Wald Test** |
| --- |
| Df Chisq Pr(>Chisq)  Food 4 68.5285 4.640e-14 ***  Sex 1 0.4473 0.5036  Food:Sex 4 36.3192 2.487e-07 ***  ---  Signif. codes: 0 ‘***’ 0.001 ‘**’ 0.01 ‘*’ 0.05 ‘.’ 0.1 ‘ ’ 1 |

| 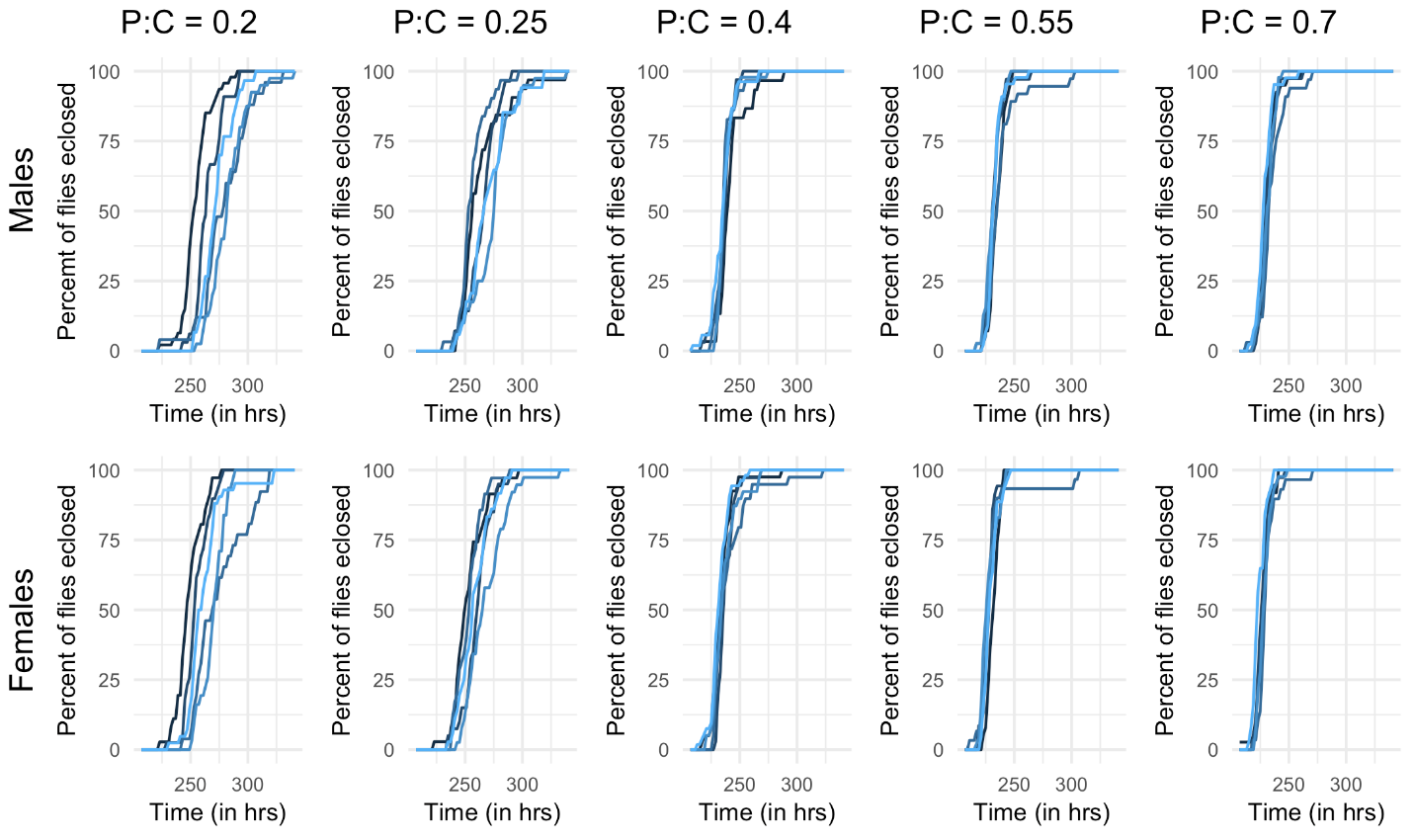 |
| --- |
| **Figure S1**. Mean percentage of flies of A) female and B) male flies that eclosed over time which were reared on different diets. Each line represents a replicate of the experiment. |


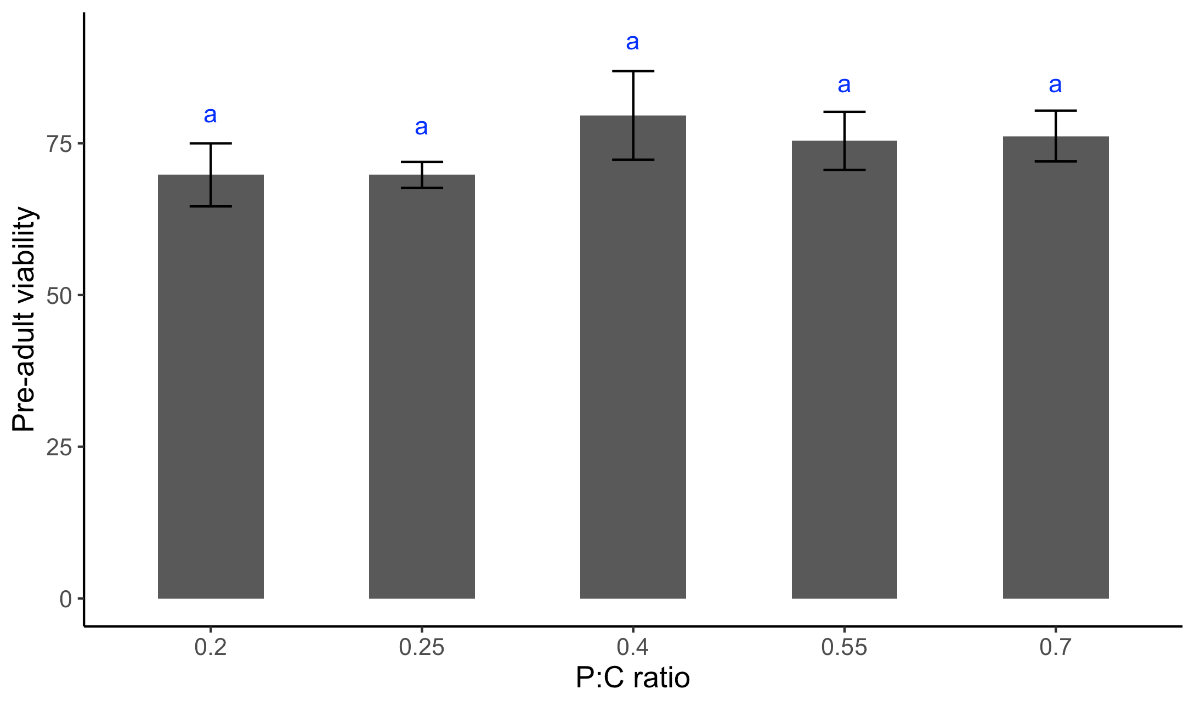


**Figure S2**. Mean (±SD) pre-adult viability of flies being reared on different diets. The bar plot represents the pre-adult viability of the flies being reared on diets with different P:C ratios. The error bar represents the standard deviation of the pre-adult viability data.


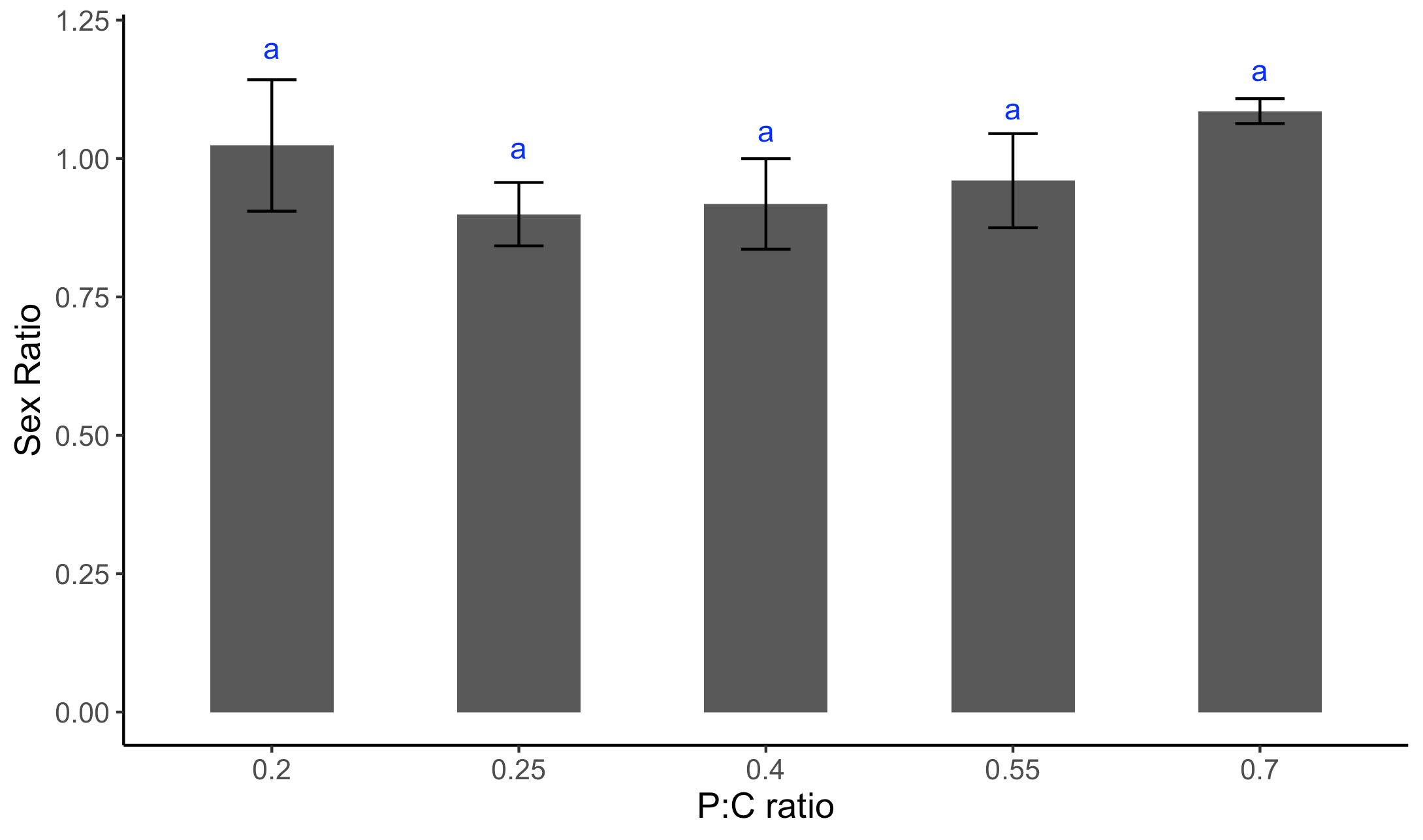


**Figure S3**. Mean (± SD) sex ratio of flies being reared on different diets. The bar plot represents the sex ratio of the flies being reared on diets with different P:C ratios. The error bar represents the standard deviation of the sex ratio data.


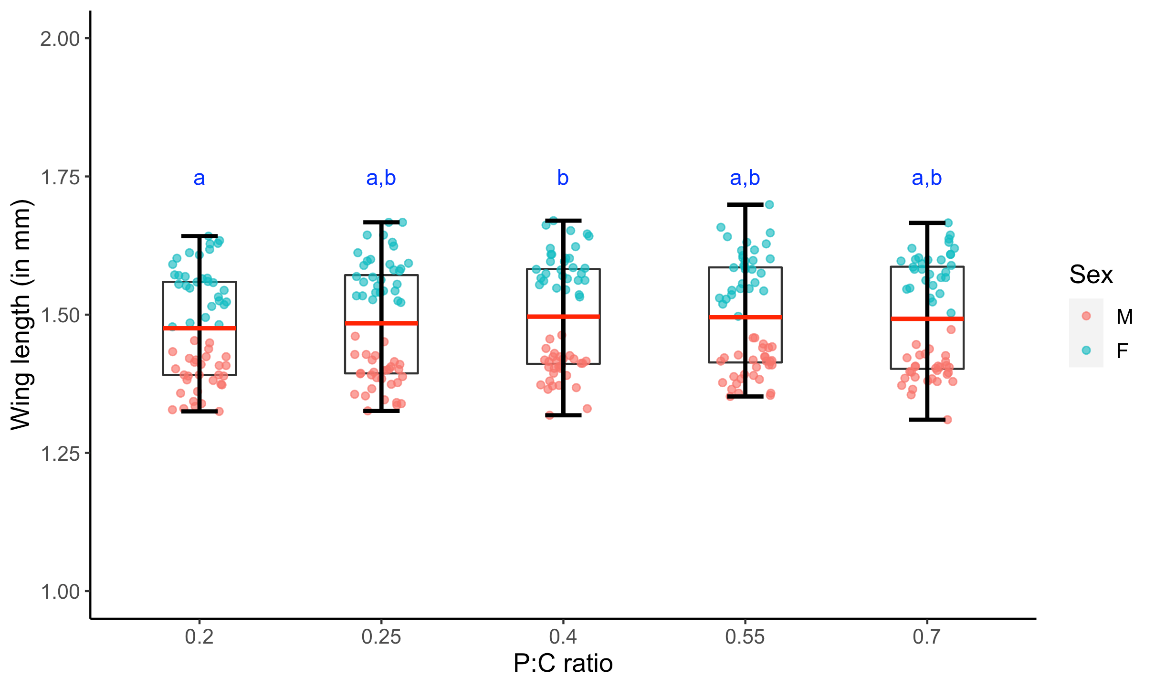


**Figure S4.** Wing length as a proxy for body size of flies that were reared on different diets. The box plot represents the wing length of the male and female flies, which were reared on diets with different P:C ratios. The bold red line represents the mean wing length of combined male and female data. The cyan and red scatter points represent wing length of the individual female and male flies, respectively.

| 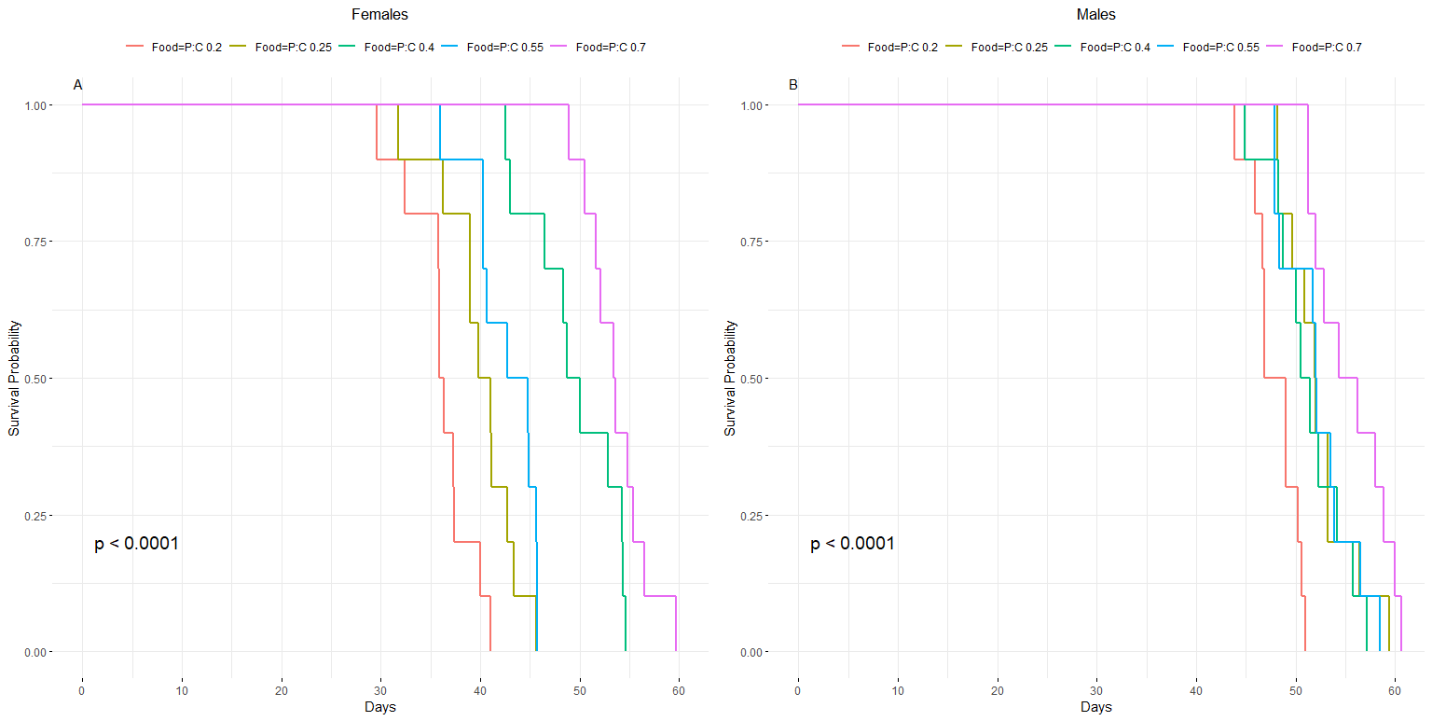 |
| --- |
| **Figure S5.** Mean survival probability of (A) female and (B) male flies over days that are reared on different diets. Each line represents the diet on which the flies were reared on. |

| 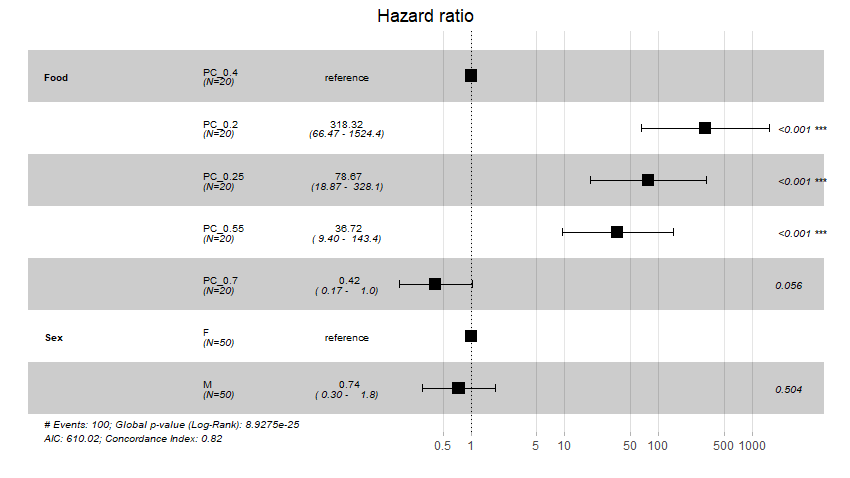 |
| --- |
| **Figure S6. Hazard ratios for the impact of diet composition and sex on lifespan.** This forest plot illustrates the hazard ratios for survival as influenced by different protein-to-carbohydrate (P:C) ratios and sex. The reference category for diet is P:C ratio of 0.4, and for sex, it is female (F). Each square represents the hazard ratio point estimate for a given category, with horizontal lines denoting 95% confidence intervals. The dashed vertical line denotes a hazard ratio of 1, indicating no effect. The numbers on the right-hand side indicates statistical significance (p-value). Statistically significant differences (p < 0.05) are marked with asterisks. The global p-value from the Log-Rank test and model fit statistics including Akaike Information Criterion (AIC) and Concordance Index are provided at the bottom of the plot. |

| 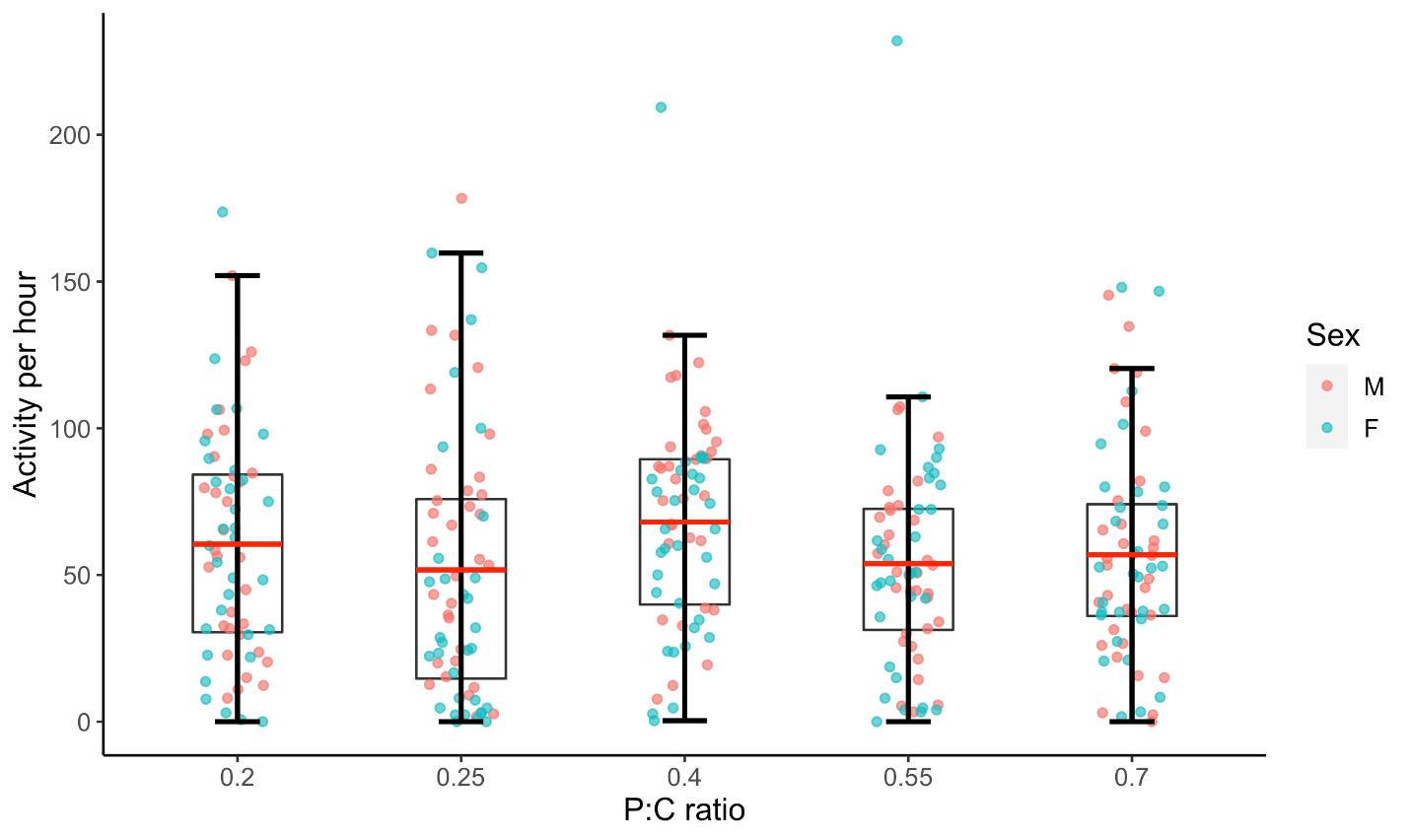 |
| --- |
| **Figure S6:** Mean (± SD) activity of flies per hour that are reared on different diets. The box plot represents the activity per hour of the male and female flies, which were reared on diets with different P:C ratios. The bold red line represents the mean activity of combined male and female data. The cyan and red scatter points represent activity of the individual female and male flies, respectively. |

| 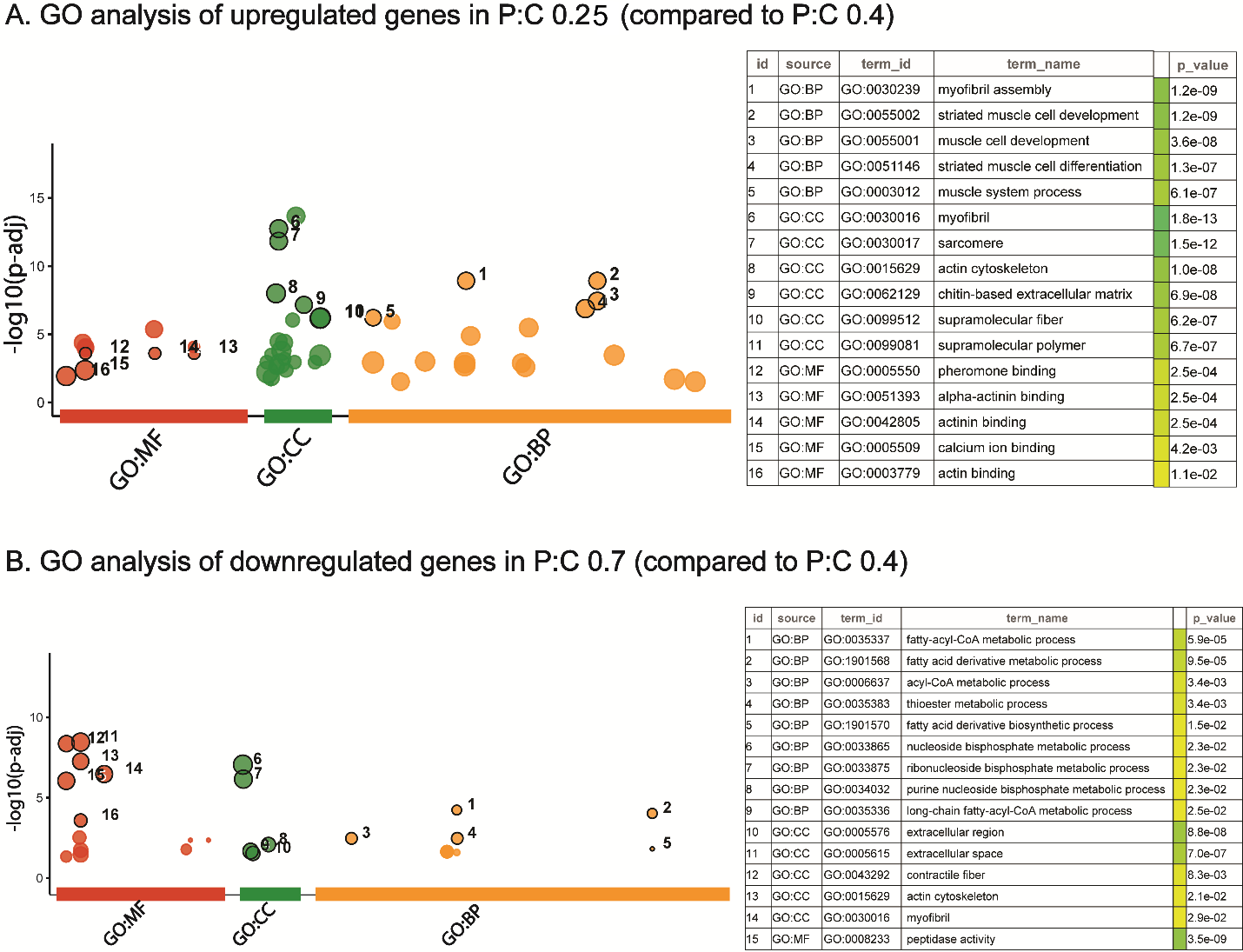 |
| --- |
| **Figure S8.** Gene Ontology (GO) analysis of gene expression in response to dietary protein-to-carbohydrate ratios. (A) Upregulated gene GO terms in P:C 0.25 vs. P:C 0.4, and (B) downregulated gene GO terms in P:C 0.7 vs. P:C 0.4 are presented here. Bubble size indicates term size. The y-axis reflects the significance (-log10(p-adj)) across biological processes (BP), cellular components (CC), and molecular functions (MF). Key enriched terms are labeled numerically and detailed in the adjacent table, which lists their GO IDs, terms, and p-values. |
